# Three-Dimensional Printed Molds for Image-Guided Surgical Biopsies: An Open Source Computational Platform

**DOI:** 10.1200/CCI.20.00026

**Published:** 2020-08-17

**Authors:** Mireia Crispin-Ortuzar, Marcel Gehrung, Stephan Ursprung, Andrew B. Gill, Anne Y. Warren, Lucian Beer, Ferdia A. Gallagher, Thomas J. Mitchell, Iosif A. Mendichovszky, Andrew N. Priest, Grant D. Stewart, Evis Sala, Florian Markowetz

**Affiliations:** ^1^ Cancer Research UK, Cambridge Institute, University of Cambridge, Cambridge, United Kingdom; ^2^ Department of Radiology, University of Cambridge, Cambridge, United Kingdom; ^3^ Department of Histopathology, Cambridge University Hospitals National Health Service (NHS) Foundation Trust, Cambridge, United Kingdom; ^4^ Department of Biomedical Imaging and Image-Guided Therapy, Medical University Vienna, Vienna, Austria; ^5^ Department of Surgery, University of Cambridge, Cambridge, United Kingdom; ^6^ Wellcome Trust Sanger Institute, Hinxton, United Kingdom; ^7^ Department of Radiology, Cambridge University Hospitals NHS Foundation Trust, Cambridge, United Kingdom

## Abstract

**PURPOSE:**

Spatial heterogeneity of tumors is a major challenge in precision oncology. The relationship between molecular and imaging heterogeneity is still poorly understood because it relies on the accurate coregistration of medical images and tissue biopsies. Tumor molds can guide the localization of biopsies, but their creation is time consuming, technologically challenging, and difficult to interface with routine clinical practice. These hurdles have so far hindered the progress in the area of multiscale integration of tumor heterogeneity data.

**METHODS:**

We have developed an open-source computational framework to automatically produce patient-specific 3-dimensional–printed molds that can be used in the clinical setting. Our approach achieves accurate coregistration of sampling location between tissue and imaging, and integrates seamlessly with clinical, imaging, and pathology workflows.

**RESULTS:**

We applied our framework to patients with renal cancer undergoing radical nephrectomy. We created personalized molds for 6 patients, obtaining Dice similarity coefficients between imaging and tissue sections ranging from 0.86 to 0.96 for tumor regions and between 0.70 and 0.76 for healthy kidneys. The framework required minimal manual intervention, producing the final mold design in just minutes, while automatically taking into account clinical considerations such as a preference for specific cutting planes.

**CONCLUSION:**

Our work provides a robust and automated interface between imaging and tissue samples, enabling the development of clinical studies to probe tumor heterogeneity on multiple spatial scales.

## INTRODUCTION

Molecular tumor profiling is used to stratify patients and identify new actionable targets for precision therapeutics. The assessment is typically based on data from a single tumor biopsy.^1^ Often, however, tumors display such a high degree of heterogeneity that a single tissue sample is insufficient to capture the full molecular landscape of the disease.^2^ A prime example of such spatial heterogeneity is renal cell carcinoma (RCC), which has been shown to be radiologically, genetically, and metabolically heterogeneous.^3-5^ Macroscopic regions with distinct genotypes can be identified within a single tumor through multiregional sampling.^3,6^ In parallel, radiologic imaging provides noninvasive, 3-dimensional (3D) information on phenotypic heterogeneity.^7,8^ The fact that RCC displays spatial heterogeneity at such disparate physical scales suggests that a combined approach to integrate the relevant data sources (ie, genomics, transcriptomics, radiomics) is needed to unravel the complexity of the disease^9^ and the genomic evolution of the tumor.^4,10-12^

**CONTEXT**

**Key Objective**
How can we accurately coregister biopsy locations and 3-dimensional (3D) imaging volumes to improve our understanding of tumor heterogeneity?
**Knowledge Generated**
We developed a computational framework to automatically design and 3D print patient-specific tumor molds that respect clinical requirements. We used the framework to generate 3D molds for 6 patients with kidney cancer and found accurate coregistration between tissue and imaging.
**Relevance**
Our work provides a robust and automated interface between imaging and tissue samples, enabling the development of clinical studies to probe tumor heterogeneity on multiple spatial scales.

The foundation of a combined analysis is the accurate spatial coregistration of imaging data and biopsies. However, typically, multiregional tumor biopsies are obtained after nephrectomy, when image guidance is no longer possible. The challenge of coregistering in vivo images to resected tumors has been addressed in other contexts. Previous solutions included holding the specimen with a cradle^13^ or solidified agar.^14^ However, these approaches had several disadvantages, including not being clinically usable or not providing accurate orientation. More recently, personalized 3D molds have been used to improve the accuracy of coregistration in prostate cancer^15-17^ and ovarian cancer studies.^18^

In RCC, however, 3D-printed molds remain comparatively underexplored,^19^ because the disease presents unique challenges. The first challenge arises from the pathology guidelines for assessment of radical nephrectomy specimens, which require optimal visualization of the renal sinus–tumor interface. The most commonly adopted initial plane of incision is along the long axis at midpoint, with further sectioning usually perpendicular to this plane.^20-22^ Thus, the sectioning planes are in general not the same as those used for imaging. An additional challenge is that pathologists need to preserve the integrity of structures that are required for staging, such as the renal vein. Finally, the specimen is often covered by perinephric fat,^23^ which further complicates the procedure and can make it impossible to identify relevant structures. Because of these restrictions, previous 3D printing–based coregistration methods for RCC either have been limited to preclinical models^24^ or have only focused on patients undergoing early-stage partial nephrectomy,^25^ in whom the fat-free resection margin can be used as a base. In addition, none of the previous methods addressed the issue of having different sectioning and imaging planes. Therefore, new methods are needed to accurately match macroscopic habitats defined by imaging to specific tissue regions. Importantly, these methods need to integrate smoothly into the clinical pathway to allow future use in clinical trials and potentially clinical practice.

Here, we report the design and implementation of an open-source computational framework to create image-based patient-specific tumor molds. The molds enable the coregistration of surgical tissue samples to presurgical multiparametric magnetic resonance imaging (MRI) in patients undergoing radical nephrectomy for suspected RCC. Our methodology is fully automated, producing ready-to-print 3D models directly from the MRI segmentation. It is also tailored for seamless integration with the clinical workflow. In particular, it can deal with any desired sectioning plane and is based on a robust landmark system that ensures accurate coregistration even in specimens obscured by a thick adipose layer. Although the framework was designed for renal cancer, it can be easily adapted to other types of solid tumors. As such, it constitutes a substantial step forward toward streamlining the creation of data sets with accurately matched imaging, histologic, and genomic data. Here, we present the computational details of the framework and validate its performance on 6 patients who underwent radical nephrectomy.

## METHODS

### Key Concepts

**FIG 1.**
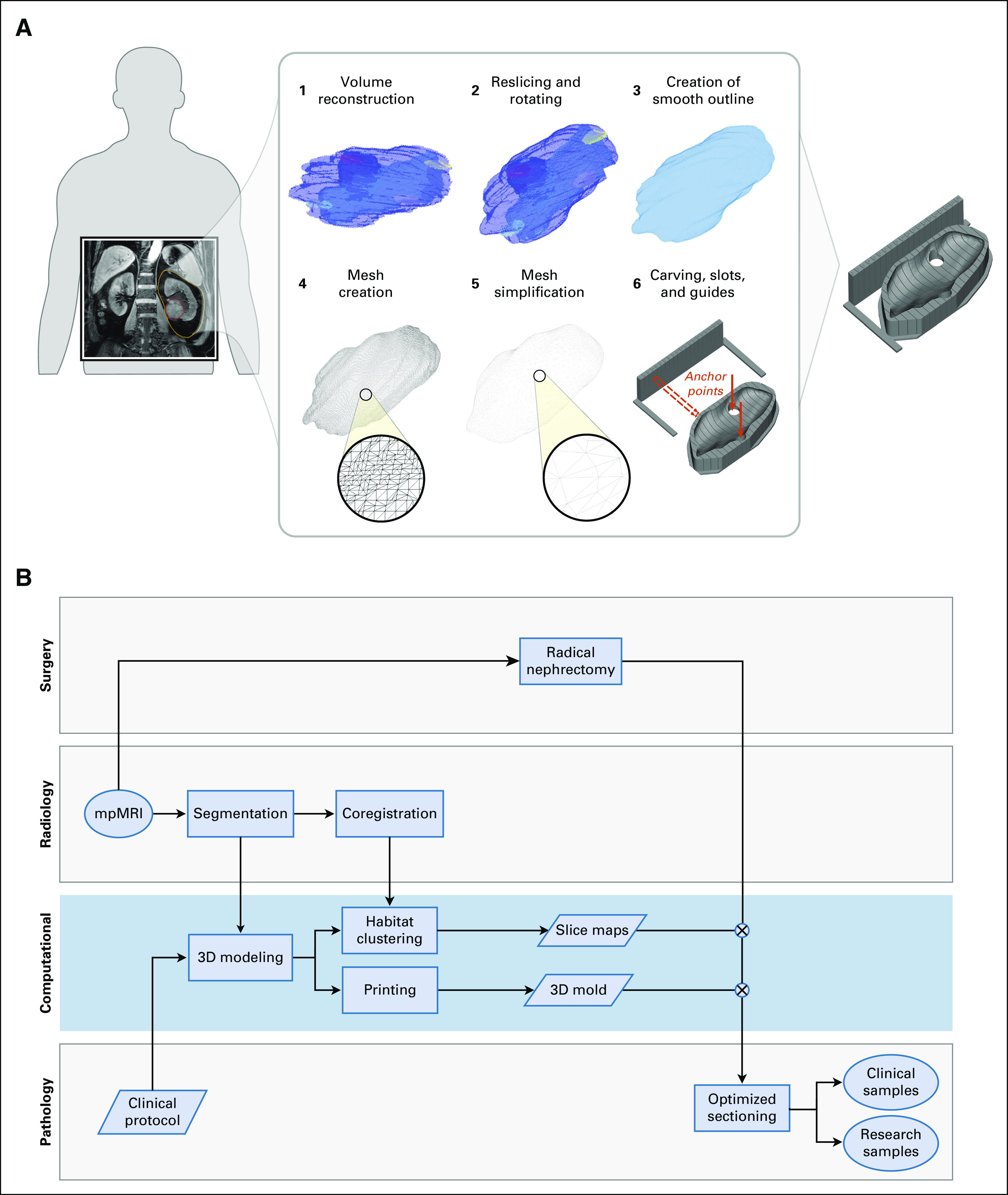
A computational framework to create image-based patient-specific tumor molds. (A) The schematic depicts the various steps of the method, bridging from magnetic resonance imaging (MRI) scans to spatially targeted surgical biopsies. The method starts with the delineation of an MRI scan, which is then reoriented, carved out of a 3-dimensional–printed mold, and used for spatially accurate surgical biopsies. The slots of the mold guide the knife for cutting. (B) Flowchart of the different analysis steps performed by the radiology, surgery, pathology, and computational groups to ensure seamless integration between the clinical and research arms. The blue box highlights the computational steps of the pipeline. mpMRI, multiparametric MRI.

We present a framework to create molds that can assist the tumor sampling process by coregistering tumor sections with MRI slices. The mold is a 3D block, with vertical slots that guide the sectioning and a cavity designed to precisely fit the resected specimen (Fig 1A). The shape of the cavity is derived from the regions of interest drawn by a radiologist on an MRI scan. The 3D modeling process involves several steps, including volume creation, reorientation, smoothing, mesh creation, and the addition of slots and guides (Fig 1A). All steps proceed automatically, and they integrate with the clinical workflow (Fig 1B). The code is available online on https://github.com/markowetzlab/cutter. See Appendix for additional methods.

### Automated 3D Modeling

#### Step 1: Image segmentation.

Our approach requires two types of regions of interest (ROIs) to be drawn on the images: tissue segmentations and anatomic landmarks. Tissue segmentations are needed to define the mold cavity and to test the spatial accuracy of the framework. They include the tumor, normal kidney, and perinephric fat. Combined, they form the global outline of the specimen, which defines the shape of the mold.

In addition, at least four anatomic landmarks are needed to determine the correct orientation of the specimen inside the mold. The first two landmarks are the upper and lower poles of the kidney, which ensure that the kidney can be sectioned along or transverse to its long axis at midpoint.^20^ The other two anatomic landmarks are the hilum (exit point of renal vessels and ureter) and the points in the tumor and/or normal kidney with the thinnest fat coverage, referred to as contact points. They are used to ensure that the specimen is accurately positioned.

#### Step 2: Image orientation.

Our approach controls the orientation of the specimen within the mold. The first orientation challenge concerns the direction along which the specimen has to be sectioned, following pathology protocols for renal cancer staging. To address this, we apply a 3D rotation to the images and create new slices that align with the preferred sectioning plane, which is defined by the tumor centroid and the upper and lower poles.

The second challenge concerns the need to accurately orient the specimen in the mold, even when it is covered in perinephric fat. We overcome this challenge by defining reference landmarks that are expected to be exposed and identifiable in the specimen and placing them at the base of the mold. These points act as anchors that ensure that the specimen is correctly positioned. The points are marked in the mold by carving 2-cm holes in the base of the mold that enable the pathologist to see and feel them (red arrows in Fig 1A). The 2 landmark points used for this purpose are the hilum and the tumor contact point.

Once the image has been rotated, we extract the outline volume needed for the mold and smooth the surface using a Gaussian kernel. The final output is a 3D integer matrix that embeds the correctly oriented volume as well as the location of the landmark points. This part of the process is implemented in MATLAB (MathWorks, Natick, MA).

#### Step 3: Mold generation and 3D printing.

The mold generation process consists of several steps (Fig 1A). First, the volumetric matrix obtained previously is converted into a mesh and then simplified by face reduction, adaptive remeshing, Laplacian smoothing, and Taubin smoothing.

Once the mesh is smooth enough for printing, it is carved off from a solid block-shaped base, and vertical slots are created to guide the knife during sectioning. In addition, a set of vertical guides is added to one side of the mold to aid with the positioning of the knife. The location of the interslot spaces in both the guides and the mold is designed to match the exact location of the imaging slices of interest. In addition, the guides are numbered such that particular slices can easily be identified and compared with imaging. Finally, we carve the reference holes at the bottom of the mold with a diameter of 2 cm at the hilum and contact landmark points. This part was implemented in Python with interfaces to Meshlab and OpenSCAD. The subsequent slicing of the 3D model was carried out with Slic3r for print preparation (Prusa Research, Prague, Czech Republic).

## RESULTS

### Ethics and Patient Cohort

The method was designed as part of a physiologic study currently being undertaken at the University of Cambridge with the aim of integrating imaging and tissue-based biomarkers to unravel tumor heterogeneity in renal cancer. Informed consent was obtained for the Molecular Imaging and Spectroscopy with Stable Isotopes in Oncology and Neurology (MISSION) substudy in renal cancer after prior approval by the East of England–Cambridge South Ethics Committee (REC: 15/EE/0378).

**FIG 2.**
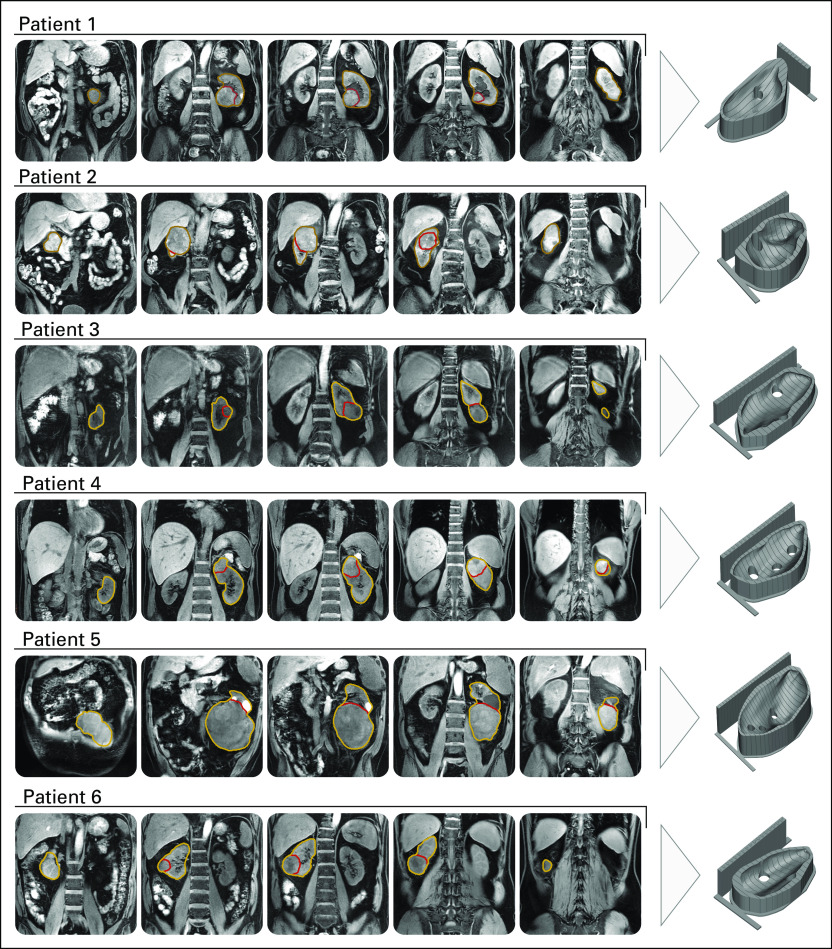
Optimized, patient-specific tumor molds. Representative T_1_-weighted magnetic resonance imaging slices and corresponding 3-dimensional renderings of the tumor molds created for the 6 patients included in the study.

Of the 6 patients included in the analysis, 5 had clear cell RCC and 1 had rhabdomyosarcoma of the kidney (Fig 2). The patient with rhabdomyosarcoma was initially included because the cancer had been diagnosed as renal cancer presurgically. Because the 3D mold printing and sectioning protocols were the same as those used for the other patients with RCC, it was decided to retain the sarcoma as a test of the generalizability of the methodology. Relevant clinical data are listed in Table 1.

**TABLE 1.**
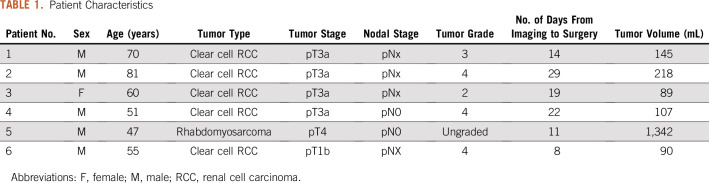
Patient Characteristics

### 3D Mold Generation and Sample Sectioning

Tumor, normal kidney, and perinephric fat were segmented manually on a presurgical T_1_-weighted MRI image, as well as the hilum, tumor and kidney contact point, and kidney poles. For the first patient, the renal pelvis was also segmented. The segmentations were checked by a radiologist with 15 years of experience in genitourinary imaging (E.S.).

We generated and 3D-printed molds for each patient using the computational framework described earlier. After discussion with the pathologist, it was decided that the first patient would be sectioned longitudinally to the kidney, whereas the other 5 were sectioned transversally.

The automated design and generation of each mold took < 5 minutes per patient. Manual verification of the segmentation and mold results took between 10 and 20 minutes. Printing each mold took between 12 and 24 hours.

The specimens were placed in the mold and sectioned 20 minutes after nephrectomy. The resection margins were inked for R staging, and all the perinephric fat was preserved. A slice where all the habitats of the tumor were present, as well as being sufficiently separated from the hilum, was chosen for sectioning in each patient. Cuts were made with a 12-inch CellPath Brain Knife (CellPath, Newtown, United Kingdom).

### Anatomic Landmark Validation

**FIG 3.**
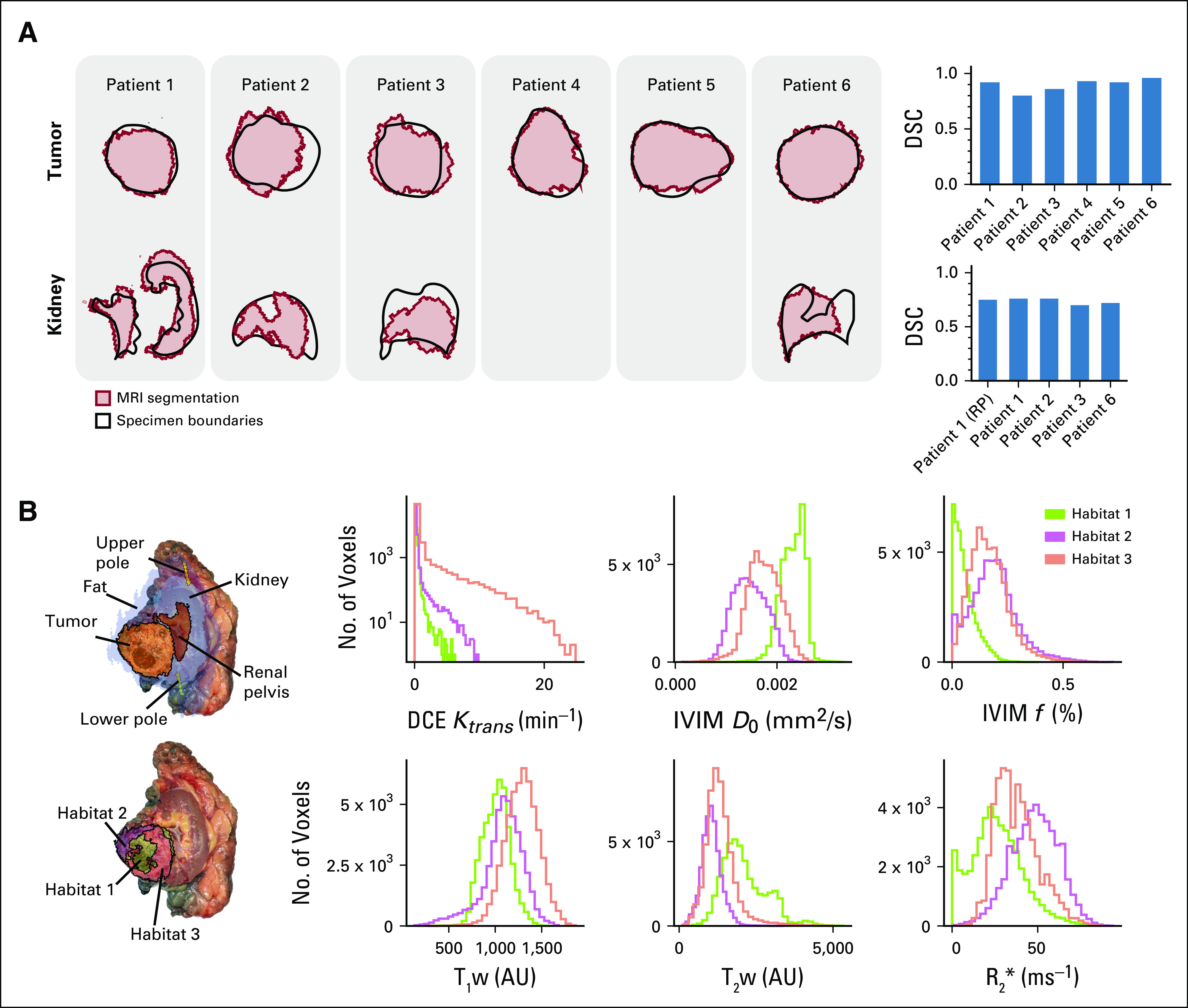
Validation results. (A) Overlay of the tissue region boundaries (black) and the corresponding magnetic resonance imaging (MRI) segmentations (red) for tumor and kidney regions. Dice similarity coefficients (DSCs) are calculated for tumor and kidney tissues separately. (B) Left: Overlay of a photograph of the section from the first patient and the corresponding MRI maps, including anatomic region segmentations (top) and multiparametric tumor habitats (bottom). Right: Relative distributions of imaging parameters for the 3 tumor habitats. AU, arbitrary units; DCE, dynamic contrast-enhanced; IVIM, intravoxel incoherent motion; RP, renal pelvis; T_1_w, T_1_-weighted; T_2_w, T_2_-weighted.

In the first patient, the selected slice resulted in a clean longitudinal cut of the kidney, including the renal pelvis, and a cross-section of the tumor, as illustrated in Figure 3A. The tumor presented two hemorrhagic areas and a necrotic core. The other 5 patients were sectioned transversally, with patients 2, 3, and 6 including large portions of normal kidney.

Each patient’s slice was placed on a flat surface and photographed. We then manually contoured the reference tissues (tumor, kidney, and renal pelvis where visible) on the tissue photograph. We coregistered the MRI segmentations and tissue contours manually, obtaining Dice similarity coefficients (DSCs)^26^ of 0.92, 0.80, 0.86, 0.93, 0.92, and 0.96 for the 6 tumor ROIs, as shown in Figure 3A. The 4 ROIs containing healthy kidney yielded DSCs of 0.76, 0.76, 0.70, and 0.72, respectively. For the first patient, the renal pelvis yielded a DSC of 0.75.

### Functional Signal Validation

Motivated by the presence of a necrotic core in the first patient, we performed an additional validation step based on the spatial distribution of different functional imaging parameters inside the tumor. Multiparametric MRI images were coregistered and used to define spatial habitats using *k*-means clustering. In particular, we used T_1_- and T_2_-weighted images, T_1_ map, $K^{\text{trans}}$ from dynamic contrast-enhanced MRI as a measure of tumor vascular leakage, the D_0_ diffusion coefficient and perfusion fraction from intravoxel incoherent motion MRI imaging (f) as a measure of cellularity and tumor perfusion, and R_2_* as a measure of oxygenation. We found 3 distinct habitats, as shown in Figure 3B.

All three habitats presented with distinct distributions with respect to perfusion fraction f, $K^{\text{trans}}$, and R_2_* maps, as shown in Figure 3B. We found habitat 1 to be poorly perfused and have a high diffusivity, T_1_-weighted hypointensity, and T_2_-weighted hyperintensity. This habitat overlapped with the necrotic area found in the resected specimen.

Habitats 2 and 3 showed similar parametric distributions. Habitat 2 was adjacent to the kidney and showed the highest levels of $K^{\text{trans}}$. Habitat 3 showed the lowest diffusion levels, as well as high R_2_*.

## DISCUSSION

Capturing the full complexity of the disease is challenging in cancers such as RCC, where tumors typically display a high degree of spatial heterogeneity both at the imaging and genomics levels. In this article, we have presented a new computational framework that overcomes a key challenge for the combined analysis of imaging and genomics data—the need to accurately match macroscopic habitats defined by imaging to specific tissue regions in an automated way and without disrupting routine clinical practices. By integrating smoothly into clinical practice, our methodology has the potential to be widely applicable in clinical trials and therefore enable the creation of unprecedented data sets with matched imaging, histologic, and genomics data.

Our framework successfully integrated all the steps to automatically produce 3D-printable molds directly from MRI segmentations. This facilitates the inclusion of mold-guided samples into clinical studies because molds can be generated fast with minimal additional workload.

Our approach was designed to address one of the limitations of previous 3D printing–based coregistration methods, which assume that tumors can be sectioned along the same plane that was used for MRI imaging. This assumption generally interferes with pathology protocols. Commandeur et al^27^ proposed a methodology to coregister histologic planes to MRI slices for prostate cancer. However, this coregistration has to be performed a posteriori, and therefore the surgical biopsies would need to be obtained without image guidance, which might result in suboptimal tumor sampling.^10^ Instead, our approach uses a landmark system based on the definition of two reference points drawn by the radiologist on the MRI scan (the upper and lower poles of the kidney). These points are then used to define the rotation to be applied to the images. We found that the rotation successfully provided the expected longitudinal or transversal cuts of the kidney.

The second challenge addressed by our approach is the presence of perinephric fat, which adds two complications to the tissue coregistration process: the difficulty in predicting the exact shape of the resected specimen, because the definition of optimal margins is controversial,^28^ and the lack of an anatomic frame of reference to correctly position the specimen in the mold. Removing or trimming the fat may interfere with clinical practice, as it could compromise the surgical margins, which need to be evaluated for the presence of tumor cells.^29^ A solution has been previously proposed for partial nephrectomies, using the inner parenchymal surface of the tumor as the base of the mold.^25^ This method involved the surgeon inserting fiducial markers into the tumor during surgery, which interrupts the routine clinical pathway. In addition, partial nephrectomy is only recommended to treat small renal masses,^30^ so patients with more advanced disease, who have typically poorer outcomes and are therefore of particular clinical relevance,^31^ would not be tractable with this approach.

Our methodology instead relies on a second set of key landmarks that can be used to orient the specimen even when there is a large component of fat. These reference points are placed at the base of the mold and marked with holes that allow the pathologist to confirm their correct positioning. This approach resulted in an accurate coregistration between imaging and resected specimen in 6 specimens corresponding to renal cancers of stages III and IV. In particular, we found that anatomic image segmentations agreed with the corresponding tissue outlines after mold-assisted sectioning, with DSCs ranging between 0.86 and 0.96 for tumor regions and between 0.70 and 0.76 for healthy kidney regions.

In addition, we observed that the tumor habitats identified from multiparametric MRI images from patient 1 coincided with observable features of the tissue. In particular, habitat 1 presented all the characteristics of necrotic tissue (poor perfusion, high diffusion, T_1_-weighted hypointensity, and T_2_-weighted hyperintensity) and, indeed, coincided with the necrotic core of the tumor.^32^ Similarly, habitat 3, which was closest to the normal kidney and therefore potentially could have better vascular access, was found to have high $K^{\text{trans}}$.

As expected, there was a thick layer of fat surrounding the specimens, which made it difficult to see the kidney or identify its orientation by simple visual inspection. This would have been a challenge even in the standard clinical setting, but the mold generally provided useful support and assistance.

Our methodology is agnostic to both the type of tumor and the imaging modality. The only input required by the computational framework is a binary mask corresponding to the volume of interest and a set of reference points that determine the preferred orientation. As an example of the generalizability of the method, we also assessed a patient who had rhabdomyosarcoma of the kidney (patient 5, Table 1), obtaining consistent accuracy values.

Our approach shares some limitations with most other coregistration approaches. First, in this study, there was a time constraint between imaging and surgery, which was independent of the mold-building process. Imaging occurred between 2 and 4 weeks before surgery, which could have resulted in anatomic changes and therefore a suboptimal mold design. However, typical tumor doubling times for renal cancer are long and suggest that the effects should be minor.^33,34^ Importantly, compared with slower manual approaches, our automated method reduces the mold design process to under half an hour, with 3D printing taking a further 24 hours at maximum. This implies that for tumors with rapid growth rates surgery and mold-guided sectioning could be performed as early as 2 days after imaging. Shape-wise, additional uncertainty may arise from the segmentation of the structures on the MRI images. Although several approaches for semiautomatic segmentation of kidney tumors exist,^35-37^ the preferred option is still manual contouring. Our methodology requires the additional delineation of perinephric fat, for which manual contouring, after discussion with the surgeon, is preferred. Although placing the point with the least fat coverage at the bottom of the mold helps reduce the uncertainty, intraoperative decisions may result in a different fat distribution. Having a single-sided mold (without an upper half) means that changes in the upper side of the specimen do not affect the accuracy, but any variations in the other half might do so. Finally, the work presented here demonstrates the robustness and spatial accuracy of the methodology, but before the technology can be routinely used in the clinic, testing over a wider patient population from a clinical trial will be required.

The methodology we have presented here will be a core element of the WIRE renal cancer trial (ClinicalTrials.gov identifier: NCT03741426). By tightly integrating into the workflow of clinical trials, our methodology will enable the creation of large, spatially matched, multiscale data sets including radiomics, genomics, and histology data. This may allow true personalized treatment decisions to be made based on imaging as a surrogate for molecular characteristics, which in the case of renal cancer would include the choice of surveillance versus surgery for small renal cancers or surveillance versus adjuvant therapy for later-stage localized RCCs.

## Figures and Tables

**TABLE A1.**
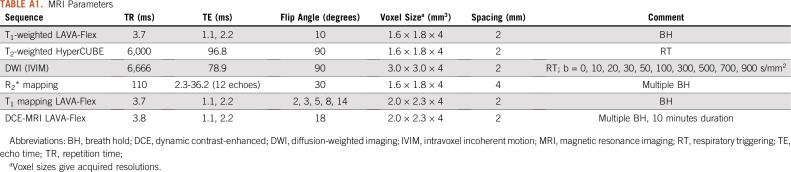
MRI Parameters

## Appendix

### Materials and Methods

#### Code.

All the code necessary to reproduce these results, including volume orientation, 3-dimensional (3D) mold design, 3D printing, and habitat generation, can be found at GitHub (https://github.com/markowetzlab/cutter).

#### Patient cohort.

All patients recruited for the MISSION study between December 2018 and January 2020, a total of 9 patients, were considered for 3D mold printing. Of those patients, 3 were excluded from the validation study presented here; 1 patient was excluded as a result of having withdrawn consent to imaging (no mold was designed), 1 as a result of having a paraganglioma (no mold was designed), and 1 as a result of not having tissue photographs for anatomic validation. The analysis presented here is based on the remaining 6 patients (Fig 2).

The methodology we have presented here will be a core element of the WIRE renal cancer trial (ClinicalTrials.gov identifier: NCT03741426). The WIRE trial is a prospective, phase II, multiarm, window-of-opportunity clinical trial with a Bayesian adaptive design. It will involve up to 76 patients with T1b+, N0-1, M0-1 clear cell renal cell carcinoma. The trial will be conducted across multiple centers in the United Kingdom. It is estimated that approximately 40 patients will be recruited in Cambridge. Mold-guided biopsies will be taken in all of the patients in Cambridge, thus providing further validation of the value of the method in a population that replicates much of the diversity of the clinical target population. If successful, the methodology will then be extended to other WIRE trial centers.

#### Magnetic resonance imaging data acquisition.

The 3D model of the tumor was designed based on a T_1_-weighted magnetic resonance imaging (MRI) scan acquired using a Dixon imaging sequence (Appendix Table A1) acquired between 2 and 4 weeks before surgery on a clinical 3T MRI (Discovery MR750; GE Healthcare, Waukesha, WI). Regions of interest (ROIs) were manually delineated by a radiologist (S.U., with 2 years of experience in genitourinary imaging) on each slice of the MRI scan using OsiriX (Version 10.0.0 [Rosset A, et al: J Digit Imaging 17:205-216, 2004]). The contours were drawn on coronal unenhanced T_1_-weighted images using registered T_2_-weighted and postcontrast T_1_-weighted images to verify the accuracy of the ROIs. The segmentation was independently reviewed by a second radiologist (E.S., with 15 years of experience in genitourinary imaging). ROIs were exported from OsiriX to comma separated value files (.csv) encoding the coordinates of the edges of the ROI on each slice using the Export ROIs plugin (Version 1.9). The centroid of each ROI was calculated as the mean of all *x*, *y*, and *z* coordinates of the voxels within it.

#### Image preprocessing.

Before generation of parameter maps, deformable motion correction was applied in MATLAB (MathWorks, Natick, MA) and using Advanced Normalization Tools within the Insight Segmentation and Registration Toolkit (ANTs/ITK) (Avants BB, et al: Front Neuroinform 8:44, 2014). In the case of diffusion-weighted imaging (DWI) MRI, this was applied across acquisitions with differing b-values; in the case of dynamic contrast-enhanced (DCE) MRI, this was applied across acquisition time points, and the associated T_1_ maps were transformed accordingly. Parameter maps were then generated using MATLAB in the case of DWI intravoxel incoherent motion (IVIM) and using MIStar (Apollo Medical Imaging Technology, Melbourne, Australia) in the case of DCE-MRI, employing the Tofts model (Tofts PS, et al: J Magn Reson Imaging 10:223-232, 1999) and a model arterial input function. R_2_* maps were generated at source on the MRI scanner using standard manufacturer software. All parameter map volumes were then aligned to the T_1_-weighted reference series used to prepare the mold. This was performed in two stages. First, each parameter map volume was resampled into the space of the T_1_-weighted reference series. Finally, and only if necessary, a rigid registration transform to more closely align the map with the reference image was determined manually using the software package ITK-SNAP; this transform was then applied to the parameter map volume using MATLAB.

#### Mold orientation.

The method proceeds as follows. First, the MRI scan is resampled to achieve an isotropic resolution of 1 × 1 × 1 mm^3^ using nearest neighbor interpolation, as implemented in CERR (Deasy JO, et al: Med Phys 30:979-985, 2003), an open source MATLAB environment for radiology research. Then, two 3D rotations are applied. Several vectors connecting the structure centroids are defined to guide the reorientation process, as follows:

$$\begin{array}{rr} v_{\text{L}} & =0.5\times (v_{\text{hilum}}+v_{\text{tumor contact}}),\\ v_{\text{LC}} & =v_{C_{0}}-v_{\text{L}},\\ v_{\text{poles}} & =v_{\text{upper}}-v_{\text{lower}}, \end{array}$$

where *V*_*C*_0__ indicates the coordinates of the centroid *C*_0_, with $v_{\text{upper}}$ representing the centroid of the upper pole and $v_{\text{lower}}$ the centroid of the lower pole. The first rotation aligns $v_{\text{poles}}$ with the *y*-axis. The second rotation is performed around the *y*-axis, aligning the x−z projection of $v_{\text{LC}}$ with the *z*-axis. Combined, the two rotations ensure that the orientation conditions are satisfied. Other rotation choices could also be easily implemented.

Before extracting and exporting the reoriented volume for mold design, the surface is smoothed using 3D Gaussian filtering with a convolution kernel of size 9 × 9 × 9 voxels and standard deviation of 3 voxels. Finally, the MRI images are sliced along the x−z plane with a spacing of 1 cm. These are used to build reference maps that will later guide the tissue sampling process; they also coincide with the location of the mold’s slots.

#### Design optimization and mold generation.

The resliced tumor segmentation was exported from MATLAB and imported into a Python script for postprocessing and mold generation. First, the marching cubes algorithm (Lewiner T, et al: J Graphics Tools 8:1-15, 2003) was applied on the 3D volume for conversion to a mesh consisting of faces and vertices. Second, to ensure integrity of the resulting tumor mesh, close vertices were merged, duplicate faces and vertices removed, faces from nonmanifold edges removed, and all face normals orientations inverted. Third, the number of faces was reduced to a maximum of 5,000 by performing quadric edge collapse decimation to simplify the mesh and reduce computational overhead. Fourth, the first smoothing step with a Laplacian kernel was performed. Fifth, because Laplacian smoothing can result in geometric issues in certain scenarios, faces were again removed from nonmanifold edges; duplicated faces and vertices were removed as well. Sixth, Taubin smoothing was performed to remove remaining irregularities. Last, remaining holes in the mesh were closed to ensure a continuous surface for mold generation and printing. Detailed parameters for each step can be found in the file *filter.mlx* on https://github.com/markowetzlab/cutter.

#### 3D printing.

The model was sliced using PrusaSlicer (Prusa Research, Prague, Czech Republic) and printed with 0.2-mm layer height on a Prusa i3 MK3 printer loaded with RS PRO PLA filament (RS Components, Corby, United Kingdom).

#### Habitat clustering.

To guide the process of tissue sampling, imaging maps were created for each tumor slice. The maps were obtained by combining multiparametric MRI images and clustering them into several spatial clusters.

Along with the reference T_1_-weighted images, additional sequences were acquired to define phenotypic habitats in the first patient. In particular, the images used for clustering were the T_1_- and T_2_-weighted images, T_1_ map, $K^{\text{trans}}$ from DCE-MRI, the diffusion coefficient and perfusion fraction from IVIM MRI imaging (f), and R_2_*. Images were obtained on a 3T MRI scanner in coronal orientation with a slice thickness of 4 mm. Scans were corrected for motion artifacts and coregistered using rigid transformations. Additional details on the images, parameter maps, and methods can be found in Appendix Table A1.

Habitats were obtained by applying *k*-means clustering on the set of coregistered images as well as the (*x,y,z*) coordinates corresponding to each voxel, to ensure spatial cohesion. The number of clusters was set to the maximum number that would allow taking 3 samples from each habitat. In practice, this translated into increasing the number of clusters until any of the habitats had an area smaller than approximately 3 cm^2^.

#### Evaluation of spatial accuracy.

The slice was placed on a flat surface and photographed. Tissue contours were drawn on the image, being completely blinded to the MRI segmentations. The resulting outline and the shape predicted after reorientation of the magnetic resonance segmentation were then overlaid and coregistered using manual rigid registration, maximizing the overlap between the tumor contours. The accuracy of slice position recovery was assessed after resection by comparing the DSC of MRI segmentations and the corresponding tissue contours. This coefficient is defined as:

$$DSC=\frac{2|X\cap^{\text{​}}Y|}{\left|X|+|Y\right|}$$

where the overlap of two binary masks *X* and *Y* (segmentations originating from different image sources) can be calculated. The higher the Dice similarity coefficient, the larger is the overlap between the two binary masks.
